# Fermentation‐Driven Valorization of Cashew Apple: Combined Influence of Terroir, Fruit Phenotype, and Clarification Level on Microbial and Biochemical Composition

**DOI:** 10.1155/ijfo/5521006

**Published:** 2026-04-26

**Authors:** Fabrice S. Codjia, Ingrid Collombel, Vincent Chochois, Jean-Christophe Meile, D. Sylvain Dabade, B. Pélagie Agbobatinkpo, Kevin Bethune, Paulin Azokpota, Joseph Dossou, Adrien Servent, Nawel Achir

**Affiliations:** ^1^ Laboratory of Food Science and Technology, Faculty of Agronomic Sciences, University of Abomey-Calavi, Abomey-Calavi, Benin, uac.bj; ^2^ QualiSud, Université de Montpellier, Avignon Université, CIRAD, Institut Agro, IRD, Université de La Réunion, Montpellier, France, univ-reunion.fr

**Keywords:** biochemistry, biotechnological valorization, cashew apple, fermentation, geographic effect, microbial diversity

## Abstract

This study provides an integrated assessment of how terroir, fruit phenotype, and clarification level jointly influence the microbial and biochemical composition during the fermentation of cashew apple, a tropical fruit with high valorization potential. Three processing matrices (pulp, juice, and clarified juice) were fermented spontaneously, and cultivable microbial diversity was identified using Nanopore sequencing, while sugars, organic acids, and polyphenols were quantified by HPLC. While fruit phenotype had no significant effect (at the 5% level) on the biochemical composition, terroir significantly affected pH, titratable acidity, condensed tannins, ascorbic acid, and flavonoid levels. Microbial community dynamics evolved over time. At 24 h, no significant differences in bacterial or fungal composition were observed across terroirs or phenotypes. However, by 48 h, bacterial diversity increased, while fungal profiles remained relatively stable. The dominant bacterial genera were *Lactiplantibacillus*, *Leuconostoc*, *Weissella*, *Acetobacter*, and *Gluconobacter*, while the predominant fungal taxa included *Hanseniaspora*, *Pichia*, and one or more genera belonging to the order *Saccharomycetales*. These findings highlight how agroenvironmental factors and minimal processing conditions can shape the fermentation process and open avenues for developing fermented cashew apple–based beverages with improved nutritional and sensory qualities.

## 1. Introduction

Spontaneous fermentations of foods and beverages result from natural microbiological transformation processes driven by complex communities of indigenous microorganisms from the environment, raw materials, or processing equipment [1]. These fermentations are valued for their ability to create unique organoleptic properties, enhance nutritional profiles, and produce functional foods with probiotic potential. However, they are also characterized by limited control over quality parameters. Variations in temperature, pH, and humidity can compromise process and product standardization [2]. This lack of regulation increases the risk of establishment and proliferation of potentially spoilage and even pathogenic microorganisms. Moreover, spontaneous fermentations may lead to the production of toxic microbial byproducts, such as mycotoxins, ethyl carbamate, and biogenic amines, which complicate safety and commercialization [3, 4]. The dynamics of spontaneous fermentation in plant‐based matrices, particularly fruits, are strongly influenced by a combination of endogenous and exogenous factors. Among the endogenous factors, fruit cultivar and phenotype inherently shape the biochemical composition (notably pigments, sugars, pH, and secondary metabolites), which in turn modulate the growth and metabolism of indigenous microbial populations [5]. Exogenous factors can be grouped into two categories: those related to the production environment and those associated with processing technologies. Several studies have shown that climate, soil type, and agroecological practices significantly affect the diversity of fruit‐associated microbiota across regions [6, 7]. Processing techniques, such as paring, crushing, and clarification, also modulate microbial ecosystem structures by selectively removing or allowing proliferation of certain species [8].

The combined analysis of terroir, phenotype, and fruit preparation level thus offers a promising approach to elucidate the variability observed in spontaneous fermentations. Thanks to advances in molecular biology, particularly microbial identification based on Nanopore sequencing, it is now possible to characterize the structure and dynamics of microbial communities as a function of terroir, fruit phenotype, and the level of clarification.

In this context, cashew apples (*Anacardium occidentale*), a highly abundant but yet underutilized agricultural by‐product of the cashew industry, represent an interesting substrate for investigation. Owing to their substantial production volume and high sugar content, cashew apples have been explored for wine, vinegar, food‐grade alcohol, and functional beverage production [9–11]. However, their pronounced astringency due to high levels of flavonoids and condensed tannins [12, 13] limits direct consumption. Fermentation can mitigate this astringency by partially degrading phenolic compounds through microbial metabolism, improving organoleptic qualities and expanding potential applications [14, 15].

Most strains used in beverage production are allochthonous to cashew apples. Yet, spontaneous fermentation of this matrix occurs within 24 h after harvest, indicating a highly active endogenous fermentative community [16]. Investigating this ecosystem holds promise for transforming the cashew apple value chain through the development of novel fermented products that can address current health and nutritional challenges. Although the effects of terroir, fruit phenotype, and clarification level on cashew apple composition have been studied separately [17, 18], and several genera such as *Pichia, Hanseniaspora, Torulaspora, Candida, Saccharomyces*, and *Acetobacter* have been identified during spontaneous fermentation [9, 19], no study to date has integrated these factors to investigate how they jointly shape the cultivable microbiota and its association with fermentation‐driven biochemical changes. This gap limits our understanding of the fermentation ecology of cashew apple, a fruit with high valorization potential. From a biotransformation perspective, this study aims to profile the cultivable microbial consortia and key biochemical traits of cashew apple fermentations, as influenced by terroir, fruit phenotype, and processing practices. The overarching objective is to identify native microbial candidates with fermentative potential to support the development of novel fermentation‐based processes and value‐added products derived from cashew apple, such as functional beverages produced through mixed fermentation.

## 2. Materials and Methods

### 2.1. Sample Collection, Processing, and Experimental Design

A total of 300 mature cashew apples (*Anacardium occidentale*), of two fruit phenotypes (red and yellow), were collected for this study, during three independent sampling campaigns conducted between March–April 2023 and March–April 2024. Harvesting was carried out randomly from multiple orchards across two terroirs (Dassa‐Zoumè in central Benin and Djougou in the northwestern region). Each terroir–phenotype combination included 25 fruits per campaign, totaling 75 samples per group across all campaigns (red—Dassa; yellow—Dassa; red—Djougou; yellow—Djougou).

Strict hygienic measures were maintained throughout the harvesting and transportation process. Cashew apples were detached manually using sanitized gloves and tools and then immediately packed on ice in sterile freezer bags. All samples were stored in coolers and transported to the laboratory within 4 to 10 h.

Upon arrival, apples were processed under aseptic conditions. Nuts were removed, and each batch of 25 apples corresponding to a given phenotype was individually ground into pulp using an electric grinder (Sokany Plug, China). From this pulp, a portion was extracted to obtain juice using an electric juicer (Kuvings, Germany). For juice clarification, a gelatin solution (2.5 mL of 0.1 g/mL food‐grade gelatin, Vahiné, France) was added per 1000 mL of juice [17].

To assess the influence of geographical origin, fruit phenotype, processing type, and fermentation time on the biochemical and microbial characteristics of cashew apple matrices, the study was structured around three main experimental components:

- Effect of terroir and phenotype on biochemical composition
 From each sample batch, deseeded apples were ground into pulp under aseptic conditions. Nonfermented pulps (0 h) were analyzed for their biochemical properties.
- Effect of terroir and phenotype on microbial composition
 Juices from the pulps were allowed to undergo spontaneous fermentation at 30°C for 24 h. Microbial analysis was then performed on these fermented juices.
- Effect of processing type and fermentation time
 A complementary experiment using only red apples from Dassa‐Zoumè evaluated the effect of processing type (pulp, juice, and clarified juice) and fermentation duration (0 h, 48 h) at 30°C. Biochemical and microbiological analyses were performed at all time intervals to monitor fermentation dynamics.

Each matrix at each time point was prepared in triplicate, placed in sterile glass jars covered with aluminum foil, and subjected to spontaneous fermentation.

### 2.2. Biochemical and Physicochemical Analyses

#### 2.2.1. pH

The pH of both clarified and nonclarified juices was measured using a glass electrode pH meter (SI Analytics, Germany). In the case of the cashew pulp, the samples were homogenized in distilled water (ratio 1:9) [20].

#### 2.2.2. Titratable Acidity

The titratable acidity of the samples was determined using a TitroLine easy automatic titrator (SI Analytics, Germany) with 0.1 N NaOH up to pH 8.2. The resulting acidity content was expressed as milliequivalents per liter.

#### 2.2.3. Dry Matter

The dry matter content of the samples was determined in accordance with the AOAC method no. 027.005 (1984).

#### 2.2.4. Determination of Total Soluble Solids

The total soluble solids of the samples were determined using a Brix meter (0–55°Brix) (Nohawk, China).

#### 2.2.5. Polyphenol Analysis

The analysis of polyphenols involved the quantification of total polyphenols, condensed tannins, and flavonoids. However, these quantifications require a prior extraction of phenolic compounds. In this study, phenolics were extracted from both the pulp and the raw cashew apple juice before analysis. In contrast, for clarified juice, the different classes of polyphenols were quantified directly, without any prior extraction step.

##### 2.2.5.1. Extraction of Phenolic Compounds

For cashew apple juices and pulps, phenolic compounds were extracted with a mix of acetone/water/formic acid (70/29/1) [21]: 7 mL of acetone, supplemented with 2.9 mL of water containing 1% formic acid, was added to 3 g of the sample. Subsequently, the mixture was subjected to ultra‐turrax (IKA, Germany) for 1 min, which facilitated disruption of the cell walls. Then, the extract was agitated for 45 min using a Reax 2 rotary homogenizer (Heidolph, Germany). The supernatant was subsequently recovered following centrifugation at 10,000 g for 10 min at 4°C. The extraction was repeated two times more. The resulting supernatants were then pooled and evaporated to dryness using a Genevac EZ HCl (Biopharma, UK). Following evaporation, the residue was recovered in 2 mL of a methanol–water mixture (1:1, v/v) and filtered through a 0.45‐μm polytetrafluoroethylene syringe filter prior to analysis.

##### 2.2.5.2. Determination of Total Polyphenols

Total polyphenols were quantified using the Folin–Ciocalteu method [22, 23]. The clarified juice samples were used directly, while the other phenolic extract, nonclarified juices and pulps, were diluted 25‐fold and 50‐fold, respectively. Quantification was conducted by combining 1 mL of the sample with 1 mL of distilled water and 5 mL of 0.2 M Folin–Ciocalteu reagent. The reaction mixture was agitated for 3 min prior to the addition of 4 mL of 7.5% sodium carbonate. After 1 h of incubation in the dark, the reaction was read at 760 nm on a Specord S600 spectrophotometer (Analytik Jena, Germany). The results were expressed as grams of equivalent gallic acid per 100 g of initial sample.

##### 2.2.5.3. Determination of Condensed Tannins

The determination of condensed tannins was conducted using the butanol/HCl [24, 25]. Two milliliters of the acetone/butanol/HCl mixture (70% acetone, 30% butanol/HCl made itself of 95% butanol and 5% HCl) was added to 250 μL of clarified juice or 500 μL of polyphenols extracts obtained from cashew apple juices and pulps. Following the addition of 200 μL of 1% ferrous chloride (FeCl_2_) in 2N hydrochloric acid, the mixture was placed in a water bath for 2 h and 15 min at 70°C. Following the incubation period, the mixture was measured at 530 nm using a Specord S600 spectrophotometer (Analytik Jena, Germany). The results were expressed as mg eq. delphinidin/100 g.

##### 2.2.5.4. Flavonoid Determination

In the case of the clarified cashew apple juices, 20 μL was directly injected into high‐performance liquid chromatography (HPLC). In the case of nonclarified juices and pulps, 20 μL of polyphenol extracts derived from cashew apples was injected into the liquid chromatography. The HPLC Agilent 1200 series (Agilent, USA) was equipped with a C_18_ 250 × 4.6 mm × 5 μm column (ACE, USA). The diode array detector was set to 280, 330, and 380 nm. The mobile phases consisted of 1% formic acid in ultrapure water (Phase A) and acetonitrile (Phase B). The flow rate was set at 0.7 mL/min, and the temperature was maintained at 30°C. The mobile phases consisted of 1% formic acid in ultrapure water (Phase A) and acetonitrile (Phase B). The flow rate was set at 0.7 mL/min, and the temperature was maintained at 30°C. The gradient was fixed at 95% A and 5% B for the initial stage, then stabilized at 14% B at 20 min, increased to 35% B at 55 min, then increased to 100% B at 57 min, and maintained for 3 min. The mobile phase was then returned to the initial condition (5% B) at 62 min and held for 10 min before the next injection. The results are expressed as grams of quercetin equivalent per 100 g.

#### 2.2.6. Carotenoid Extraction and Identification

The extraction of carotenoids was conducted by mixing 4 g of pulp or 8 g of cashew apple juice with 20 mL of an ethanol/hexane solution (4:3, v/v) [26]. The residue was then separated from the liquid phase by filtration through a glass filter funnel (porosity no. 2) and washed twice with ethanol/hexane (30 mL) for the pulps and once for the juices. Subsequently, the liquid phase was transferred to a separating funnel and 5 mL of 10% NaCl was added, after which the aqueous layer was decanted and discarded. The organic phase was recovered, dried with anhydrous sodium sulfate, and evaporated to dryness under vacuum at 32°C using an RV 10 evaporator (IKA, Germany). The resulting carotenoid dry extract was then dissolved in 1 mL of a mixture of methyl tert‐butyl ether (MTBE) and methanol (80:20, v/v) and subsequently analyzed by HPLC.

The identification of carotenoids was conducted via HPLC using an 1100 HPLC‐DAD system (Agilent, USA). The separation of carotenoids was realized using a C_30_ column (250 × 4.6 mm × 5 μm) (YMC, Germany) equipped with a guard column. The mobile phase consisted of H_2_O as Eluent A, methanol as Eluent B, and MTBE as Eluent C. The operating temperature was 25°C, the flow rate was 1 mL/min, and the injection volume was 20 μL [27]. β‐Carotene was detected at 450 nm, and the results were expressed as the sum of the E and Z isomers.

#### 2.2.7. Extraction and Determination of Sugars and Organic Acids

The extraction was performed using an ultrasonic bath [27]. One hundred milligrams of freeze‐dried cashew apple powder was combined with 1.5 mL of acidified distilled water (H_2_SO_4_) (0.002 M, 70°C). After 15 min in an ultrasonic bath, the suspension was mixed for 2 h and centrifuged for 10 min at 15,000 rpm. The resulting supernatant was filtered through a 0.45‐μm syringe filter (Sigma‐Aldrich, USA), after which it was transferred to an HPLC vial for subsequent analysis.

The cashew apple juices were subjected to direct analysis after centrifugation at 15,000 rpm and filtration through a 0.45‐μm filter (Sigma‐Aldrich, USA) without further extraction. The concentrations of organic acids (acetic, lactic, and ascorbic) and sugars (glucose, fructose, and sucrose) were determined by HPLC using an Agilent System model 1100 equipped with Shodex SUGAR (300 mm × 4.6 × 5 μm) (Shodex, Japan). The solvent was a solution of 0.01% H_2_SO_4_, and the flow rate was 1 mL/min in isocratic mode. Detection was set at 245 nm. Organic acids were analyzed by UV and sugars by refractometer (RA). All molecules were quantified using external standards.

### 2.3. Microbial Diversity Analyses

#### 2.3.1. Microbial Count

Cashew apple pulp, nonclarified juice, and clarified juice samples were suspended in physiological saline water at a ratio of 0.1% (w/v). Microbial enumeration was performed using appropriate 10‐fold serial dilutions. Total mesophilic aerobic bacteria were enumerated on the surface of Plate Count Agar (PCA) plates (OXOID CM0325, United Kingdom) and incubated at 30°C for 72 h. Lactic acid bacteria (LAB) were enumerated on the surface of Man, Rogosa, and Sharpe (MRS) agar (CM0361 Oxoid, United Kingdom) at pH 6.2. The Petri dishes were incubated under anaerobic conditions at 30°C for 48 h. Yeasts were enumerated on the surface of Sabouraud chloramphenicol agar (Biokar BK021HA, France) at 25°C for 72 h. Acetic acid bacteria were enumerated on the surface of Kneifel medium at 30°C for 48 h. Preparation of 1 L of this medium required 30 g of yeast extract (Biokar Diagnostics A1202HA, France), 20 g of bacteriological agar (Biokar Diagnostics A1010HA, France), and 1 mL of a 2.2% (w/v) ethanolic solution of bromocresol green (Sigma‐Aldrich, USA). After sterilization, 20 mL of ethanol, 100 mg/L of cycloheximide (Sigma‐Aldrich, USA), and 12.5 mg/L of penicillin (Sigma‐Aldrich, USA) dissolved in ethanol were added.

#### 2.3.2. Microbial Identification

##### 2.3.2.1. DNA Extraction

After numeration, only Petri dishes containing between 10 and 200 colonies were retained for further analysis. This selection range corresponds to the counting thresholds used to ensure reliable quantification, yielding approximately 3.0 to 9.9 log CFU/g for bacteria and 3.2 to 9.5 log CFU/g for yeasts, depending on the dilution factors applied, regardless of cashew apple phenotype, region, or matrix type. All colonies from each selected plate were aseptically harvested and suspended in 2 mL of isotonic saline solution. Bacterial isolates obtained from MRS, Kneifel, and PCA media were pooled into a single suspension for bacterial identification, while colonies recovered from Sabouraud chloramphenicol agar were used exclusively for fungal identification. All suspensions were stored at −20°C for subsequent taxonomic identification by Nanopore sequencing.

The DNA was extracted from the samples using the FastDNA SPIN Kit protocol (MP Biomedicals, USA), with the addition of enzymatic lysis steps with RNase A (50 μg) and Proteinase K (1 mg) between Steps 4 and 5 of the commercial protocol. The elution volume was 80 μL.

The double‐stranded DNA concentration was measured through fluorimetric quantification with the dsDNA Broad Range Assay Kit on a Qubit instrument (Thermo Fisher Scientific, USA). Following measurement, sample concentrations were adjusted to ensure a concentration range of 0.1 to 2 ng/μL for all samples.

Because DNA was extracted from pooled colonies isolated on selective media, the sequencing output reflects a qualitative presence/absence profile of cultivable taxa. This approach does not allow inference of relative abundance nor detection of noncultivable microorganisms.

##### 2.3.2.2. DNA Amplification, Library Preparation, and Sequencing

The method was based on the recommendations provided with the SQK‐LSK114 kit (Oxford Nanopore Technologies, Oxford, UK). For each DNA sample extracted previously, two distinct polymerase chain reactions (PCRs) were performed, targeting the 16S ribosomal RNA gene for bacterial DNA (ca. 1550 bp) [28] and the internal transcribed spacer (ITS) region (400–1000 bp) [29] for fungal DNA. The primer pairs used included adaptor sequences at their 5′ ends (underlined bases below) to facilitate the incorporation of synthetic barcodes during a secondary PCR, allowing for sample multiplexing. The primer pairs were as follows:

• 16S_forward primer: TTTCTGTTGGTGCTGATATTGCAGAGTTTGATCMTGGCTCAG
• 16S_reverse primer: ACT​TGC​CTG​TCG​CTC​TAT​CTT​CCG​GTT​ACC​TTG​TTA​CGA​CTT
• ITS forward primer: TTT​CTG​TTG​GTG​CTG​ATA​TTG​CTC​CGT​AGG​TGA​ACC​TGC​GG
• ITS reverse primer: ACT​TGC​CTG​TCG​CTC​TAT​CTT​CTC​CTC​CGC​TTA​TTG​ATA​TGC

The PCR mixture consisted of Phusion Flash High‐Fidelity PCR Master Mix (Thermo Fisher Scientific, USA), 0.25 μM each primer, and 20 to 378 ng for bacterial DNA (mean = 61 ng). All samples were quantified as around 100 ng was required for 16S Barcoding Kit (SQK‐16S024), 0.1 to 0.9 ng was required for yeast DNA (mean = 0.37 ng), and only 1 over 3 series was quantified using the template in a final volume of 20 µL. The PCR protocol involved an initial denaturation at 98°C for 2 min, followed by 25 cycles of denaturation at 98°C for 15 s, primer hybridization at 55°C for 30 s, and extension at 72°C for 30 s, with a final extension step at 72°C for 5 min.

Subsequently, the PCR products were purified using AMPure XP magnetic beads (Beckman Coulter, Brea, California, USA) with a ratio of 0.6:1 for 16S amplicons and 0.8:1 for ITS amplicons.

Quantification of PCR products was performed for library standardization before performing a secondary PCR to incorporate known synthetic barcodes from the EXP‐PBC001 Kit (Oxford Nanopore Technologies, Oxford, UK). PCR products were then purified and quantified to adjust a balanced loading of each barcode into the libraries. Two distinct libraries were created (one for 16S and one for ITS). Libraries were then end‐repaired and ligated to Nanopore specific adapters with reagents from the NEBNext Companion Module (New England Biolabs, USA). Once purified and normalized, libraries were loaded on MinION flow cells (2x Flongles R9 (ref = FLO‐FLG001) plus 1x FlowCell R9 (ref = FLO‐MIN106) with Nanopore Ligation kit (ref = SQK‐16S024) for 16S sequencing & 3x Flongles R10 (ref = FLO‐FLG114) with Nanopore barcoding and ligation kits (respective refs = EXP‐PBC001 and SQK‐LSK114) for ITS sequencing).

#### 2.3.3. Bioinformatics Analyses

Bioinformatics analyses were conducted using the Core Cluster resources of the Institut Français de Bioinformatique (IFB; ANR‐11‐INBS‐0013). Initial processing of raw sequencing reads involved basecalling with Guppy (v6.5.7‐gpu; Oxford Nanopore Technologies) using the superior‐accuracy models provided by the manufacturer. Adapter trimming and demultiplexing, utilizing barcode kits SQK‐16S024 for 16S rRNA reads and EXP‐PBC001 for ITS reads, were also performed with Guppy. Subsequent analysis of 16S rRNA data followed the EMU workflow [30], employing the GROND database (version GTDB207nr) [31] for taxonomic classification. ITS data were analyzed using a modified version of the NANOCLUST pipeline [32, 33], with taxonomic assignments made against the UNITE database (v10.0). For downstream diversity analyses, compositional data were rarefied to an even depth of 19,949 reads per sample. These rarefied data were then converted to qualitative (presence/absence) data by applying a relative abundance threshold of ≥ 5% per sample. Beta diversity was assessed using the Jaccard index, suitable for binary data. The significance of observed differences in community composition between experimental groups was tested using permutational multivariate analysis of variance (PERMANOVA) with 99,999 permutations. Beta diversity and composition analyses were performed in R (v 4.4.3) using packages phyloseq (v 1.46.0) and microViz (v 0.12.4).

### 2.4. Statistical Analysis

Statistical analysis, except for sequencing‐related data analysis and bioinformatics, was performed using XLSTAT software (XLSTAT‐Basic, Addinsoft, France). Student’s *t*‐tests were conducted to compare group means, with statistical significance set at *p* < 0.05.

## 3. Results and Discussion

### 3.1. Effect of Geographical Origin on the Biochemical Composition of Nonfermented Cashew Apple Pulp

The terroir effect was studied by analyzing nonfermented cashew apple pulp from two localities: Dassa and Djougou, regardless of the phenotype (Table 1). Statistical analysis revealed no significant differences between pulps from the two regions in terms of dry matter content, °Brix, fructose, and glucose levels. The measured values were consistent with those previously reported in the literature [34, 35].

**TABLE 1:** Biochemical parameters of nonfermented cashew apple pulps from 2 different geographical origins (regardless of the phenotype).

Region	TS (%)	TTA (meq/L)	pH	°Brix %	Polyphenols (g/kg)	Tannins (mg/kg)	Flavonoids (mg/kg)	Carotenoids (mg/kg)	Ascorbic acid (g/kg)	Fructose (g/kg)	Glucose (g/kg)
Djougou	13.03 ± 0.82	0.04 ± 0.01	4.20 ± 0.13	11.08 ± 0.82	6.74 ± 2.58	112.09 ± 33.50	23.65 ± 9.14	3.63 ± 1.20	4.67 ± 0.19	37.45 ± 3.69	23.03 ± 3.15
Dassa	12.34 ± 1.86	0.03 ± 0.01	4.56 ± 0.33	10.75 ± 1.05	4.79 ± 1.29	62.85 ± 10.33	12.02 ± 2.63	3.60 ± 0.75	4.02 ± 0.67	37.97 ± 7.66	23.83 ± 4.10
*p* value	0.42	**0.04**	**0.03**	0.56	0.12	**0.01**	**0.01**	0.93	**0.03**	0.88	0.73

*Note:* Values are expressed as the means of at least three measurements per condition (*n* = 6). Statistical significance was determined at *p* < 0.05, with differences considered significant when *p* values were below this threshold. The bold values correspond to *p* values and indicate statistically significant results (*p* < 0.05).

On the contrary, a significant difference was observed in the following parameters: titratable acidity, pH, ascorbic acid, and also tannins and flavonoids, between the two regions. Djougou cashew apples exhibited higher acidity levels (40 vs. 30 meq/L), lower pH (4.20 vs. 4.56), and higher ascorbic acid levels (4.67 vs. 4.02 g/kg) which are consistent results. Ascorbic acid levels were 10‐fold higher than the concentration found in citrus fruits [36]. Although total polyphenol contents were not significantly different, cashew apples from Djougou contained nearly twice the concentration of tannins (112.09 vs. 62.85 mg/kg) and flavonoids (23.65 vs. 12.02 mg/kg), compared to those from Dassa. This significant difference can be explained by environmental factors related to climate, soil, and surroundings. The higher concentration of tannins, flavonoids, and organic acids in cashew apples from Djougou than Dassa could be attributed to the response of cashew trees to climatic stress [37]. Indeed, Djougou is known for its harsh Sudano‐Guinean climate, characterized by a long dry season (from mid‐October to mid‐April) and a long rainy season (from mid‐April to mid‐October) [38]. The pH, which was significantly higher in Dassa compared to Djougou, reflecting lower acidity levels in the apples from Dassa, may be explained by the milder climate in Dassa. It is also a Sudano‐Guinean climate type, but with two rainy seasons (main rainy season: March to July; minor rainy season: October to November) and two dry seasons (minor dry season: August to September; main dry season: December to March) [39]. Our findings are consistent with those of Dabonne et al. [18], who reported significant differences in parameters such as pH, tannins, flavonoids, and ascorbic acid between cashew apples from three different climatic regions of Côte d’Ivoire (Zanzan, Gbèkè, and Marahou). Moreover, cashew apples from Yamoussoukro and Korhogo (two regions of the Côte d’Ivoire) exhibited significant differences in flavonoid content [40].

### 3.2. Effect of Phenotype on the Biochemical Composition of Nonfermented Cashew Apple Pulp

The effect of phenotype on biochemical composition was studied by analyzing nonfermented cashew apple pulp of red and yellow phenotypes, regardless of the terroir (Table 2). Statistical analysis revealed no significant difference in the measured parameters between yellow and red apples. Our results can be explained by the fact that both red and yellow cashew apples collected in the field were of the same physiological quality (fully ripe). According to Agbangnan et al., the juice from red cashew apples would exhibit significantly higher total soluble solids compared to that from yellow cashew apples. However, reported practices among cashew apple juice producers reveal no distinction in the use of the two cashew apple phenotypes. In Côte d’Ivoire, a study evaluating the biochemical parameters of juice from red and yellow cashew apples found significant differences in pH, total soluble solids, dry matter, total phenolic compounds, carotenoids, flavonoids, and ascorbic acid [41]. The discrepancy between our results and those reported in the literature could be attributed to the processing method of samples. In our study, biochemical parameters were measured in cashew apple pulp, whereas the literature reports measurements conducted on cashew apple juice. The extraction and filtration processes of the juice could significantly influence the biochemical parameters. During filtration, some biochemical compounds may be retained in the pomace. Additionally, variations in environmental conditions between Benin and Côte d’Ivoire also contribute to the differences observed in our findings.

**TABLE 2:** Biochemical parameters of nonfermented cashew apple pulps from 2 different phenotypes (regardless of the terroir).

Phenotype	TS (%)	TTA (meq/L)	pH	°Brix %	Polyphenols (g/kg)	Tannins (mg/kg)	Flavonoids (mg/kg)	Carotenoids (mg/kg)	Ascorbic acid (g/kg)	Fructose (g/kg)	Glucose (g/kg)
Red	12.90 ± 1.61	0.04 ± 0.01	4.34 ± 0.35	10.77 ± 0.70	6.52 + 2.61	102.52 ± 42.51	19.78 ± 9.46	3.58 ± 1.18	4.30 ± 0.85	37.89 ± 5.76	23.23 ± 3.83
Yellow	12.48 ± 1.31	0.03 ± 0.01	4.42 ± 0.29	11.06 ± 1.14	5.00 ± 1.55	72.43 ± 18.24	15.90 ± 8.54	3.65 ± 078	4.38 ± 0.64	37.54 ± 6.25	23.64 ± 4.22
*p* value	0.63	0.48	0.68	0.60	0.24	0.14	0.47	0.88	0.82	0.92	0.86

*Note:* Values are expressed as the means of at least three measurements per condition (*n* = 6). Statistical significance was determined at *p* < 0.05, with differences considered significant when *p* values were below this threshold.

### 3.3. Culturable Microbial Flora of Spontaneous Fermented Cashew Apple Juice: Influence of Geographical Origin and Phenotype

The analysis of the culturable microbial flora (≥ 5% relative abundance) of cashew apple juice spontaneously fermented for 24 h was conducted to assess the influence of geographical origin (Dassa and Djougou) (Figure 1) and fruit phenotype (yellow and red) (Figure 2). Regarding the fungal flora (Figures 1(a) and 2(a)), yeasts belonging to the genus *Hanseniaspora*, notably *Hanseniaspora* sp. and *Hanseniaspora opuntiae*, were predominant in fermented juices from both regions and across both fruit phenotypes. This genus, widely documented in natural fruit fermentations, thus appears to play an important role in the fermentation process of cashew apple juice. The recurrent presence of the *Hanseniaspora* genus, regardless of geographical origin or fruit phenotype, confirms their predominant ecological role in the spontaneous fermentation of sugar‐rich tropical fruits. Their occurrence on cashew apples may also be linked to their natural habitat in tropical regions such as Mexico, Brazil, Ghana, and Hawaii [42]. Their metabolic capabilities, particularly the production of aromatic compounds (such as acetate esters), confer significant technological importance in shaping the sensory profile of fruit juices, especially that of cashew apple juice [43].

FIGURE 1Fungal and bacterial diversities of the cultivable microbiota in cashew apple juice fermented for 24 h from the Dassa and Djougou regions. (a) Qualitative taxonomic composition based on presence/absence data. Principal coordinates analysis (PCoA) ordinations of beta diversity, computed using Jaccard distance matrices to reflect qualitative dissimilarities among fungal (b) and bacterial (c) diversities.

**Figure:**
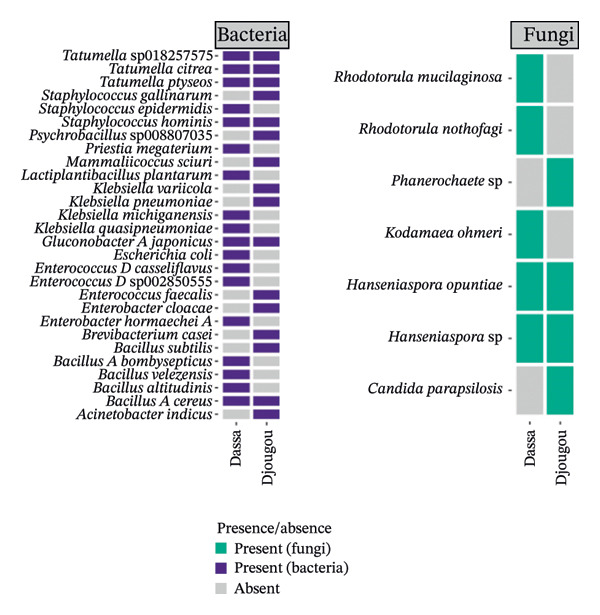
(a)

**Figure:**
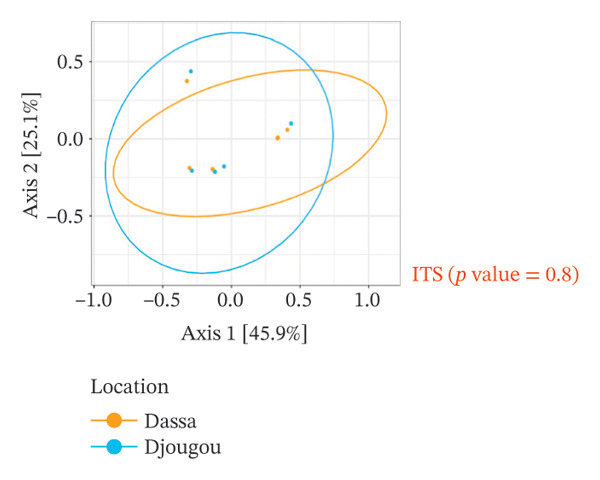
(b)

**Figure:**
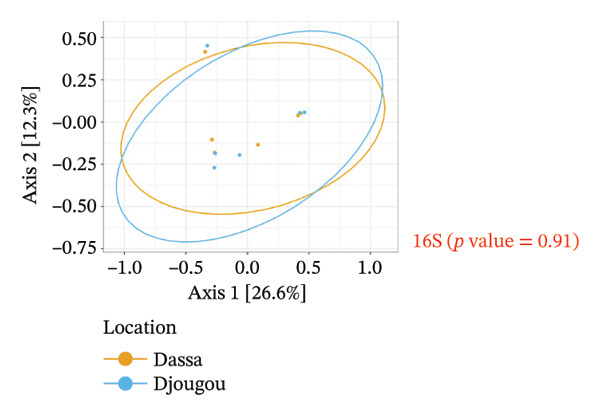
(c)

FIGURE 2Fungal and bacterial diversities of the cultivable microbiota in cashew apple juice fermented for 24 h in the yellow and red phenotypes. (a) Qualitative taxonomic composition based on presence/absence data. Principal coordinates analysis (PCoA) ordinations of beta diversity, computed using Jaccard distance matrices to reflect qualitative dissimilarities among fungal (b) and bacterial (c) communities.

**Figure:**
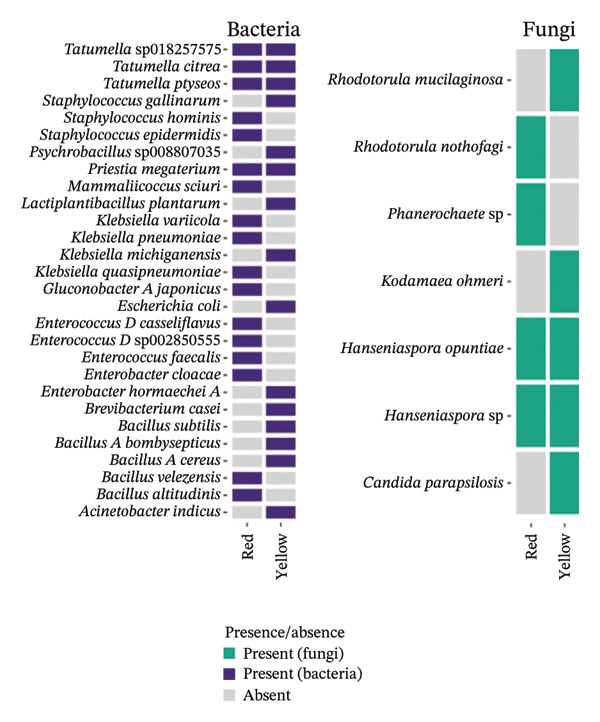
(a)

**Figure:**
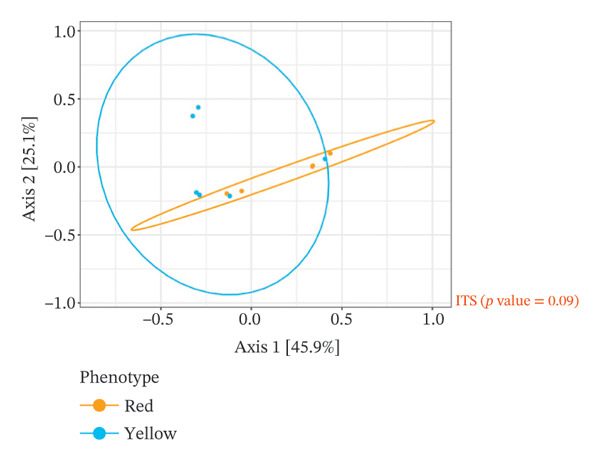
(b)

**Figure:**
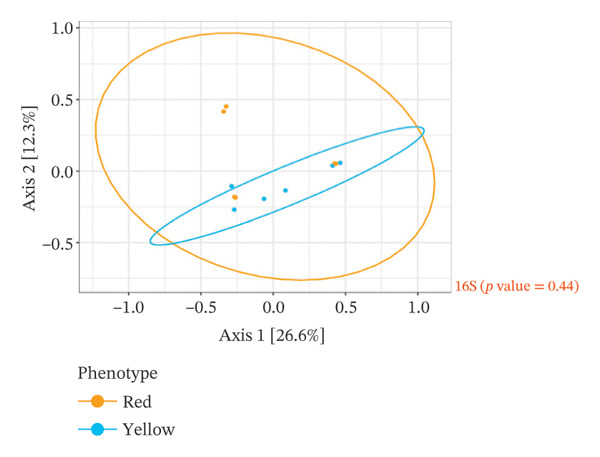
(c)

As for the bacterial flora (Figures 1(a) and 2(a)), several species were identified in samples from both Dassa and Djougou, including *Tatumella* genus (*Tatumella* sp018257575, *Tatumella ptyseos* and *Tatumella citrea)*, *Gluconobacter_A japonicus*, *Staphylococcus hominis*, and *Bacillus_A cereus*. The latter two genera may be introduced through human‐associated contamination during handling or processing. *Tatumella* was also detected in juices derived from both red and yellow phenotypes as well as the *Priestia* genus. The uniformity observed in bacterial communities across regions and phenotypes could probably be attributable to microorganisms of environmental origin [44–47]. Their presence in spontaneously fermented cashew apple juice may be explained by the absence of a washing step prior to processing. *Tatumella* and *Priestia* both well known for their associations with plants, fruits, and soils are typical examples of such environmental microbiota [48].

Beta diversity analysis (Figures 1(b), 1(c), 2(b), and 2(c)) using PERMANOVA revealed no significant differences between yeast and bacterial communities in juices from Dassa and Djougou *p* = 0.80 for yeasts; *p* = 0.91 for bacteria). Similarly, no significant differences were observed in yeast (*p* = 0.09) or bacterial (*p* = 0.44) communities based on fruit phenotype.

The identification of *Lactiplantibacillus* and *Gluconobacter* in the samples is of significant technological interest. These genera are commonly involved in liquid food fermentations, where they contribute to acidification, the production of volatile compounds, and the microbiological stabilization of beverages [49, 50]. Finally, the detection of potential opportunistic pathogenic bacteria such as *Enterococcus*, *Klebsiella*, and *Staphylococcus* genera (Figures 1(a) and 2(a)) may be associated with extrinsic factors, such as cattle grazing in certain cashew plantations. This observation highlights the need for strict sanitary oversight and improved control of processing conditions to mitigate potential health risks for consumers.

## 4. Selection of Cashew Apple Phenotype and Region for Further Studies

Based on the physicochemical results, no significant phenotypic differences were observed between unfermented yellow and red cashew apples, and only a limited regional effect was detected. Specifically, cashew apples from Djougou differed only by exhibiting higher levels of titratable acidity, condensed tannins, and flavonoids compared to those from Dassa. Furthermore, analysis of bacterial and fungal community diversity in fermented juices from both regions and both phenotypes revealed no significant differences. Based on these observations, the remainder of the study focuses on the impact of processing conditions on red cashew apples from Dassa.

### 4.1. Physicochemical Composition of Dassa Red Cashew Apple Products Obtained From Various Processing Methods Before and After 48‐h Fermentation

#### 4.1.1. Effect of Unit Operations and Processing Methods Before Fermentation

The biochemical composition of cashew apple processed into pulp, juice, and clarified juice is presented in Table 3. A significant effect of the processing method was observed on dry matter content, with a reduction of approximately 35% following juice extraction and clarification. This loss in dry matter may be attributed to the removal of cashew apple fibers during the successive unit operations involved in juice extraction and clarification. With regard to the reducing sugars analyzed, fructose was found to be predominant over glucose, regardless of the type of cashew apple product [17, 51]. The significantly higher concentrations of glucose, fructose, and ascorbic acid in the juice compared to the pulp and clarified juice can be primarily attributed to the high water solubility of these compounds and their localization within cellular vacuoles. During mechanical extraction, these hydrophilic substances are readily released into the liquid phase, while only trace amounts remain trapped within the fibrous matrix of the pulp. Furthermore, clarification with gelatin not only removes suspended solids, including polyphenolic compounds, but also eliminates part of the dissolved compounds associated with the colloidal phase, thereby contributing to a reduction in sugar and vitamin C content [17, 52].

**TABLE 3:** Biochemical parameters of three cashew apple–derived products (pulp, juice, and clarified juice) before fermentation.

Cashew‐ products	TS (%)	TTA (meq/L)	pH	derived°Brix (%)	Polyphenols (g/kg)	Tannin (mg/Kg)	Flavonoids (mg/kg)	Carotenoids (mg/kg)	Ascorbic acid (g/kg)	Fructose (g/kg)	Glucose (g/kg)
Pulp	12.88b ± 2.28	0.03a ± 0.00	4.50a ± 0.45	10.83a ± 1.03	5.02b ± 1.59	64.36c ± 4.33	13.71c ± 1.52	3.67b ± 0.83	3.57a ± 0.22	38.52a ± 7.72	24.06a ± 4.90
Juice	12.12b ± 0.69	0.03a ± 0.01	4.54a ± 0.18	10.71a ± 0.48	3.74ab ± 1.38	21.35b ± 2.25	9.13b ± 0.45	3.16b ± 001	5.02b ± 0.06	63.7b4 ± 7.78	36.83b ± 4.14
Clarified juice	8.32a ± 1.03	0.02a ± 0.01	4.09a ± 0.31	9.06a ± 0.92	1.25a ± 0.38	5.25a ± 4.05	3.51a ± 1.14	NA	3.57a ± 0.21	42.19a ± 2.29	24.89a ± 0.87
*p* value	**0.02**	0.57	0.26	0.07	**0.02**	**< 0.0001**	**< 0.0001**	**< 0.0001**	**< 0.0001**	**< 0.001**	**0.01**

*Note:* Values are expressed as the means of at least three measurements per condition (*n* = 3). Statistical significance was determined at *p* < 0.05, with differences considered significant when *p* values were below this threshold. The bold values correspond to the *p* values and indicate statistically significant differences (*p* < 0.05). Different letters (a, b, c) indicate statistically significant differences between groups (*p* < 0.05).

Titratable acidity, pH, and Brix content remained unchanged regardless of the processing technique. However, clarified juice differed significantly from the pulp in terms of secondary metabolites. Notably, substantial losses of 75%, 90%, 75%, and 100% were recorded for total phenolic content, tannins, flavonoids, and carotenoids, respectively. These reductions in secondary metabolites and dry matter from pulp to clarified juice may be attributed to the sedimentation of bioactive compounds induced by food‐grade gelatin, combined with the filtration process used during clarification. Therefore, it would be relevant to explore alternative clarification methods with lower impact on the removal of secondary metabolites, beyond the use of gelatin. Additionally, the recovery of bioactive compounds from the residues generated during clarification (sediments) could be considered in future studies for potential nutritional applications.

#### 4.1.2. Effect of Fermentation

The physicochemical composition of three fermented Red Cashew apple–derived products was compared before and after 48‐h fermentation (Figure 3). The end of fermentation was positively correlated to titratable acidity and acetic acid and negatively correlated to reducing sugars (fructose and glucose) for all three products. This can be explained by the microbial conversion of sugars into organic acids during fermentation. Several bacterial genera may be responsible for this conversion, most likely including *Gluconobacter*, *Acetobacter*, and *Lactiplantibacillus* [53].

FIGURE 3Physicochemical composition of red cashew apples from Dassa as a function of fermentation time (0–48 h) and product type (a) pulp, (b) juice, and (c) clarified juice. Each plot displays the evolution of total soluble solids (°Brix), titratable acidity (meq/L), pH, ascorbic acid content (g/kg), condensed tannins (mg/kg), and total flavonoids (mg/kg). Data are presented as mean ± standard deviation from three biological replicates (*n* = 3).

**Figure:**
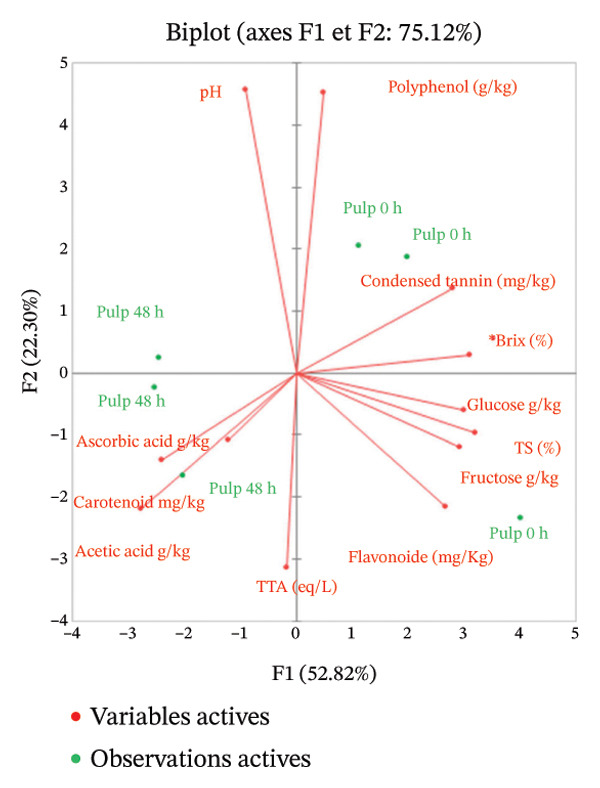
(a)

**Figure:**
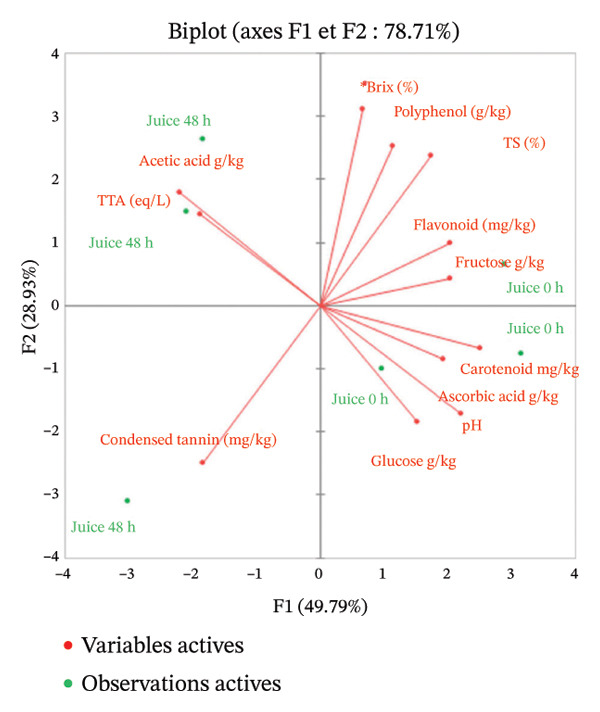
(b)

**Figure:**
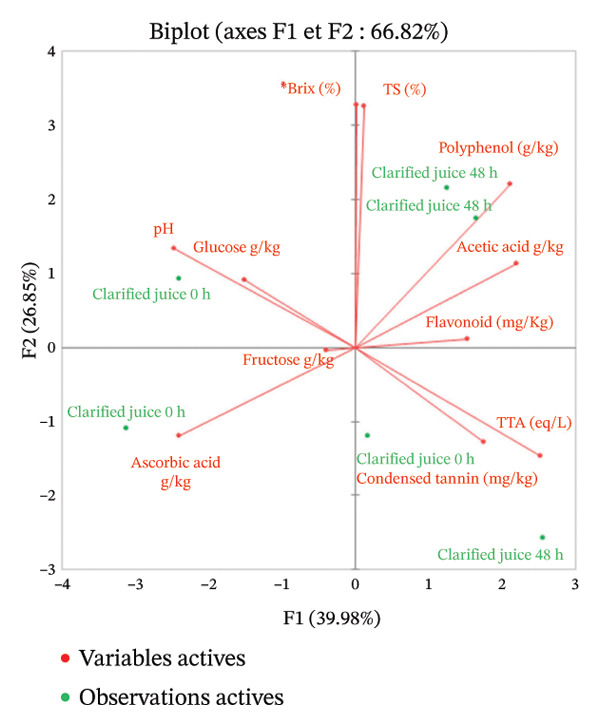
(c)

Moreover, the pulp and juice samples after 48 h of fermentation exhibited a negative correlation with total phenolic compounds, tannins, and flavonoids, suggesting microbial degradation or structural modification of polyphenolic compounds. Several bacteria, particularly those of the genus *Lactiplantibacillus*, possess enzymes such as phenol oxidases and laccases, which enable them to degrade polyphenolic compounds. This degradation could lead to an organoleptic improvement of cashew apple during fermentation thanks to this astringency reduction [54]. This phenomenon was not observed in the clarified juices, likely because the polyphenolic compounds prone to degradation during fermentation had already been removed during clarification.

In addition, except in the pulp, ascorbic acid levels decreased during fermentation. This compound appeared to be more sensitive in filtered or clarified juices, where its degradation was more pronounced.

### 4.2. Effect of the Processing Method on Microbial Diversity

#### 4.2.1. Effect of the Processing Method on Bacterial Diversity

Beta diversity analysis revealed that the observed variation in community composition could not be significantly explained by product type (pulp, juice, or clarified juice) (*p* = 0.13). This may be due to the fact that although processing methods may alter certain physicochemical parameters of cashew apple (such as condensed tannins, flavonoids, and carotenoids) through filtration and clarification, they do not create sufficiently strong selective conditions to significantly impact bacterial composition. Therefore, the same bacteria can persist regardless of the applied treatment. Since the processing unit operations did not significantly affect bacterial diversity, it is likely that the opportunistic contaminant bacteria found in both the juice and clarified juice did not originate from processing equipment or operator handling. Instead, they may have derived from the cashew orchards and the surrounding environment during cashew apple production.

#### 4.2.2. Effect of the Processing Method on Fungal Diversity

Statistical analysis to compare samples using PERMANOVA showed that the processing method (pulp, juice, or clarified juice) did not significantly affect fungal community diversity, as evidenced by a *p* value of 0.06. Although not significant, a trend of the processing method on fungal diversity is observed (*p* = 0.06, close to 0.05). This trend should be further explored in future studies with a larger sample size to confirm these results. A possible reason is that unit operations like filtration in the case of clarified juice can modulate yeast composition. Larger yeast species may become trapped in the filtration residues, which could explain the absence of several yeasts (*Starmerella etchellsii*, *Saccharomycetales* sp., *Pichia* sp.) in the clarified juice.

### 4.3. Effect of Fermentation on the Cultivable Microbial Flora Diversity of Dassa Red Cashew Apples

#### 4.3.1. Cultivable Microbial Species Uniquely Observed Before Fermentation

**FIGURE 4:**
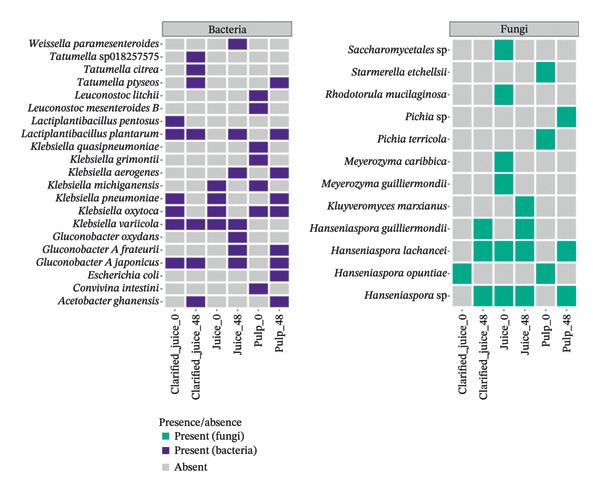
Fungal and bacterial diversities of the cultivable microbiota of red cashew apples from Dassa before (0 h) and after 48‐h fermentation under three product types.

Seven bacterial species belonging to four different genera (*Lactiplantibacillus pentosus, Leuconostoc mesenteroides_B, Leuconostoc litchii, Klebsiella michiganensis, Klebsiella quasipneumoniae, Klebsiella grimontii*, and *Convivina intestini*) were exclusively detected prior to fermentation, regardless of the cashew apple processing method used (Figure 4). Similarly, four fungal species from three genera (*Saccharomycetales* sp.*, Rhodotorula mucilaginosa, Meyerozyma guilliermondii,* and *Meyerozyma caribbica*) were exclusively detected in cashew apple juice, while two others (*Starmerella etchellsii* and *Pichia terricola*) were found exclusively in the pulp before fermentation. The presence of these species can be explained by their common involvement in the early stages of fermentation, where they are likely replaced by other species better adapted to the evolving fermentation conditions, such as changes in pH, alcohol concentration, and microbial competition for nutrients and space [55].

#### 4.3.2. Cultivable Microbial Species Exclusively Detected at the End of Fermentation

Six bacterial genera comprising nine species (*Weissella paramesenteroides*, *Tatmella sp018257575*, *Tatmella citrea*, *Tatmella ptyseos*, *Klebsiella aerogenes*, *Gluconobacter oxydans*, *Gluconobacter_A frateurii*, *Escherichia coli*, and *Acetobacter ghanensis*) and three yeast species (*Pichia* sp., *Kluyveromyces marxianus*, and *Hanseniaspora guilliermondii*) were detected only after 48 h of fermentation, regardless of the cashew apple processing method used (Figure 4). The presence of these species may indicate their adaptation to evolving fermentation conditions, particularly to increased acidity and alcohol concentrations. For instance, *A*. *ghanensis*, *Glu*. *oxydans*, and *Glu*.*_A frateurii* being acetic acid bacteria, their abundance suggest that the fermentative medium already contains alcohol to be converted into acetic acid in the presence of oxygen.

To add up, statistical analysis (PERMANOVA) of bacterial community composition before and after 48‐h fermentation revealed that the fermentative process dramatically and significantly impacted the bacteriome (*p* < 0.05), with an R‐squared value of 0.09. Unlike bacteria, yeast diversity does not change significantly between 0 and 48 h (*p* value = 0.43). This result may be attributed to the dominance of resilient yeast species capable of withstanding the fermentation conditions, and the absence of significant environmental changes likely limited the emergence or decline of specific taxa.

#### 4.3.3. Correlation Between Physicochemical Profile and Microbial Communities

**FIGURE 5:**
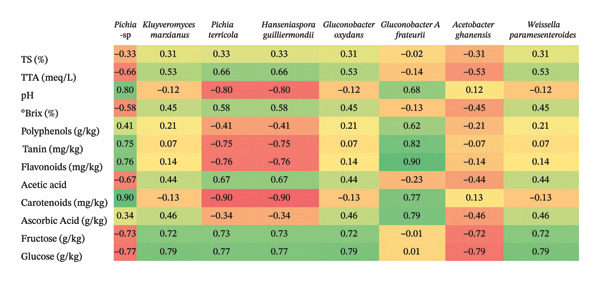
Association between chemical parameters (concentrations) and strain occurrence (presence or absence) (*n* = 3).

A multiple linear regression (OLS) was used to assess the association between the continuous outcome (chemical data of the pulp, juice, and clarified juice obtained from red cashew apples from Dassa) and a set of continuous and binary explanatory variables (coded 0/1 to indicate the presence or absence of the strain) after 48 h of fermentation (Figure 5).

Among the microbial species identified at 48 h of fermentation, *Kluyveromyces marxianus, Weissella paramesenteroides*, and *Gluconobacter oxydans* stood out due to their notable positive correlations with key compounds such as fructose [56], glucose, °Brix, and certain antioxidant molecules, including flavonoids and ascorbic acid [57]. These findings suggest a potential ability of these microorganisms to preserve specific nutritional and sensory attributes of the beverage, while simultaneously limiting the excessive consumption of fermentable sugars. These characteristics are particularly relevant for the development of mildly fermented, functional [58], and nutritionally balanced beverages.

Conversely, yeast species such as *Pichia terricola* and *Hanseniaspora guilliermondii* exhibit strong negative correlations with phenolic compounds. This likely reflects a more pronounced fermentative activity directed toward the degradation of these molecules, suggesting that these microorganisms may possess tannase activity. Given that the astringency of cashew apple is a major barrier to its consumption, *P. terricola* and *H. guilliermondii* emerge as promising candidates for reducing cashew apple astringency through targeted fermentation processes. However, the findings of the present study contrast with previous reports indicating that *Pichia terricola* (formerly known as *Issatchenkia terricola*) was positively correlated with phenolic compounds in fermented lemon juice [59]. This discrepancy may be attributed to differences in the fermentation matrix, as cashew apple and lemon juice differ markedly in their initial phenolic profiles, pH, and sugar composition. Moreover, in the context of spontaneous fermentation, interactions between *Pichia terricola* and other microbial species may alter its phenolic‐related metabolic profile. A similar phenomenon has been reported for *Hanseniaspora guilliermondii* during wine cofermentation, where interspecies interactions significantly modulated its phenolic metabolism [60].

Among acetic acid bacteria, *Gluconobacter A frateurii* exhibited a negative correlation with acetic acid production, while simultaneously maintaining positive associations with ascorbic acid and polyphenols. This dual profile mild acidification coupled with partial preservation of antioxidant compounds may be leveraged in the development of functional beverages with enhanced sensory brightness and health‐oriented properties. Recent studies have reported that *Gluconobacter A frateurii* is an exopolysaccharide (EPS)‐producing bacterium with antioxidant properties [61]. This species could be considered for inclusion in mixed‐culture fermentation systems due to its specific role in EPS production. In contrast, the weak correlation of *Acetobacter ghanensis* with pH further supports its role in contributing to the acidification of the final product.

Overall, these findings confirm that the selection of microbial strains can significantly influence the physicochemical composition of cashew apple–based fermented beverages. Strain selection thus represents a critical lever for modulating the organoleptic, nutritional, and functional properties of the final product.

## 5. Conclusion

This study highlights the diversity of the cultivable microbiota of cashew apples during spontaneous fermentation. Taking into account terroir, phenotype, and processing methods, we observed that microbial composition remains stable across these parameters but evolves over time, particularly within bacterial communities. Additionally, biochemical analyses reveal the production of organic acids and a reduction in polyphenol content after 48 h of fermentation, emphasizing the impact of the fermentation process on the chemical composition of cashew apples and the potential modulation of their sensory properties. A further perspective of this work would be therefore to carry out volatile organic compounds (VOCS) and sensory analysis with the different populations or mixed populations.

Although culture‐dependent long‐read sequencing does not provide quantitative insights into relative abundance, it offers a rapid and reliable overview of the cultivable microbial community. This qualitative identification is essential for selecting indigenous yeasts, LAB, and AAB to design relevant mixed‐culture consortia for cashew apple fermentation.

The identification of key microbial genera such as *Lactiplantibacillus, Leuconostoc, Weissella, Acetobacter*, and *Gluconobacter* among bacteria, and *Hanseniaspora*, *Pichia*, along with one or more unidentified genera within the order *Saccharomycetales* among yeasts offers interesting opportunities for biotechnological applications. A better understanding of the metabolism and interplay between these microorganisms during the fermentation process may open the prospect of the development of novel cashew apple–based products, such as the creation of healthy fermented beverages, thus valorizing a currently underutilized resource. Thus, this study serves as a reference point for exploring the technological potential of the cashew apple microbiota and highlights the relevance of controlled spontaneous fermentation in a sustainable and innovative agri‐food approach.

## Funding

This research was funded by the Partenariats Académiques Afrique‐France (PeA) programme and was implemented by the Agence Nationale de la Recherche (ANR), BIOVALOR project n°ANR‐21‐PEA2‐0006.

## Conflicts of Interest

The authors declare no conflicts of interest.

## Data Availability

The data supporting the findings of this study are available from the corresponding author upon reasonable request.
